# Chemical Elicitor-Induced Modulation of Antioxidant Metabolism and Enhancement of Secondary Metabolite Accumulation in Cell Suspension Cultures of *Scrophularia kakudensis* Franch

**DOI:** 10.3390/ijms17030399

**Published:** 2016-03-18

**Authors:** Abinaya Manivannan, Prabhakaran Soundararajan, Yoo Gyeong Park, Byoung Ryong Jeong

**Affiliations:** 1 Division of Applied Life Science (BK21 Plus), Graduate School, Gyeongsang National University, Jinju 660-701, Korea; abinayamanivannan@gmail.com (A.M.); prabhakaran.s.bioinfo@gmail.com (P.S.); 2 Institute of Agriculture and Life Science, Gyeongsang National University, Jinju 660-701, Korea; ygpark615@gmail.com; 3 Research Institute of Life Science, Gyeongsang National University, Jinju 660-701, Korea

**Keywords:** acacetin, antioxidant enzymes activity, elicitation, free radicals, secondary metabolites, cell culture

## Abstract

*Scrophularia kakudensis* is an important medicinal plant with pharmaceutically valuable secondary metabolites. To develop a sustainable source of naturaceuticals with vital therapeutic importance, a cell suspension culture was established in *S. kakudensis* for the first time. Friable calli were induced from the leaf explants cultured on a Murashige and Skoog (MS) medium containing 3.0 mg·L^−1^ 6-benzyladenine (BA) in a combination with 2 mg·L^−1^ 2,4-dichlorophenoxy acetic acid (2,4-D). From the callus cultures, a cell suspension culture was initiated and the cellular differentiation was investigated. In addition, the effect of biotic elicitors such as methyl jasmonate (MeJa), salicylic acid (SA), and sodium nitroprusside (SNP) on the accumulation of secondary metabolites and antioxidant properties was demonstrated. Among the elicitors, the MeJa elicited the accumulation of total phenols, flavonoids, and acacetin, a flavonoid compound with multiple pharmaceutical values. Similarly, the higher concentrations of the MeJa significantly modulated the activities of antioxidant enzymes and enhanced the scavenging potentials of free radicals of cell suspension extracts. Overall, the outcomes of this study can be utilized for the large scale production of pharmaceutically important secondary metabolites from *S. kakudensis* through cell suspension cultures.

## 1. Introduction

Plant tissue culture based *in vitro* culture systems acts as an alternative method for the production of secondary metabolites, thereby conserving the natural sources [1]. Plant cell cultures are considered a promising source for the sustainable production of pharmaceutically valuable secondary metabolites of industrial importance, but the commercialization of the cell-suspension-mediated synthesis of secondary metabolites is limited by low yield and poor quality [2,3]. Thus, the accumulation of high secondary metabolite contents is essential for the commercial exploitation of cell suspension cultures. In order to address the productivity issues, incorporation of elicitors in suspension cultures is claimed to be the efficient approach [4]. Although various elicitation strategies are available, the enhancement of bioactive compounds using chemical elicitors is predominantly considered because of its high efficiency and yield [4]. In general, the chemical elicitors employed predominantly belong to the plant signaling molecules such as methyl jasmonate (MeJa), salicylic acid (SA), and nitric oxide (NO) generating molecules like sodium nitroprusside (SNP). MeJa is a ubiquitous plant signaling messenger involved in diverse regulatory functions such as defense response against wounding, pathogens, temperature and salinity stress [5]. Moreover, the MeJa is a well-known elicitor of secondary metabolism in plants. Similarly, SA acts as a vital stress signaling component widely studied for its involvement in systemic acquired resistance (SAR) against diseases and pathogens [5]. In addition, NO is a free radical with multiple physiological implications in plants. According to previous studies, NO is believed to play important roles in signal transduction and plant defense by enhancing the secondary metabolism [6]. Thus these chemical elicitor molecules have been previously employed to stimulate the production of various secondary metabolites, such as isoflavonoid [7], terpenes [8], phytosterols [9], and phenylpropanoids [10]. Although the effects of MeJa, SA, and NO on the accumulation of secondary metabolites have been studied extensively, their influences are species-specific. Therefore, it is important to select the appropriate elicitor for large-scale production of secondary metabolites. Hence, in this study the effect of MeJa, SA, and NO were investigated in the cell suspension culture of *Scrophularia kakudensis*.

*Scrophularia kakudensis* Franch is a pharmaceutically important plant species distributed around the mountains of Korea, Japan, and China. Previous reports suggested the occurrence of vital secondary metabolites such as acacetin [11] and scrophulasaponins [12]. Besides the therapeutic properties, conventional propagation of the plant has been hindered by seed dormancy and narrow environmental adaptations which obstructs the utilization of the plant material. Recently, we have established an efficient micropropagation protocol for *S. kakudensis* and also investigated the content of acacetin on different tissues in the micropropagated plants [13]. Among the *Scrophularia* species, generation of callus has been described in *S. nodosa* [14] and a recent report by Khanpour-Ardestani *et al.* [15] has dealt with the cell suspension culture of *S.* *straiata*; however, there is no information available with respect to *S. kakudensis*. Owing to the medicinal importance of *S. kakudensis*, the present endeavor has established cell suspension cultures and investigated the effects of different chemical elicitors on the accumulation of bioactive compounds and elucidated the free radical scavenging properties of the extracts derived from cell cultures.

## 2. Results

### 2.1. Establishment of Cell Suspension Culture and Morphological Observation of Cells

**Figure 1:**
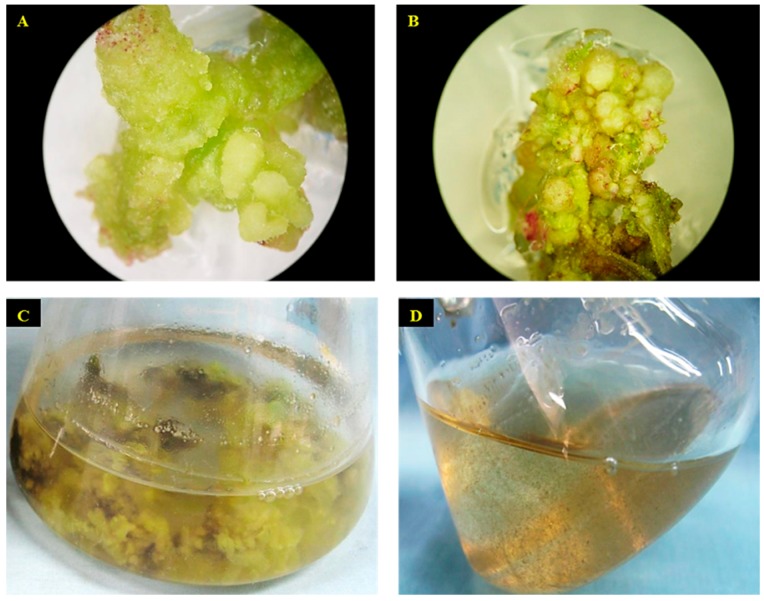
Induction of friable callus after two weeks (**A**) and four weeks (**B**); friable callus cultured in liquid medium with cells deposited on the walls of the flask (**C**); cell suspension cultures derived from callus cultures (**D**).

After two weeks, the friable calli started to emerge on the cut ends of the leaf explants cultured on the MS medium containing 3.0 mg·L^−1^ 6-benzyladenine (BA) along with 0.5 mg·L^−1^ 2,4-dichlorophenoxy aceticacid (2,4-D) (Figure 1A,B) and no callus formation was noticed in the leaf explants cultured on the medium devoid of plant growth regulators (PGRs). The callus formed was repeatedly subcultured to maintain the stock cultures. After six subcultures (each at a 25 days interval), the friable calli were transferred to the liquid MS medium containing PGRs (Figure 1C). In the initial stages the suspended calli produced clumps of cells within 10 days of inoculation and attained a homogenous granular stage after three weeks (Figure 1D). The aggregates of cells were separated by continuous shaking and the suspension of loose cell aggregates was obtained.

**Figure 2:**
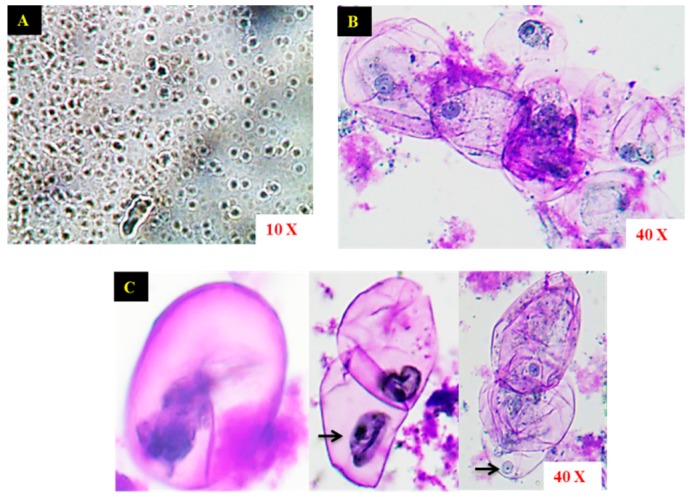
Microscopic observation of matrix of cells in cell suspension cultures (**A**); cluster of cells stained using toluidine blue (**B**); ovoid shaped cells with distinct nucleus represented with the arrows (**C**).

The initial stages of suspension culture consisted of a matrix of cells, and upon repeated passaging steps, the distinct single cells were visualized under a light microscope (Figure 2A). The microscopic observation of the suspension culture illustrated the structural characteristics of the cells is shown in Figure 2B. The morphological appearances of the cells were small, round, or oval shaped with a distinct cell wall. The concentrated nuclei of the cells were found to be bounded by the cell wall (Figure 2C).

### 2.2. Effect of Chemical Elicitors on the Contents of Endogenous Free Radicals

**Figure 3:**
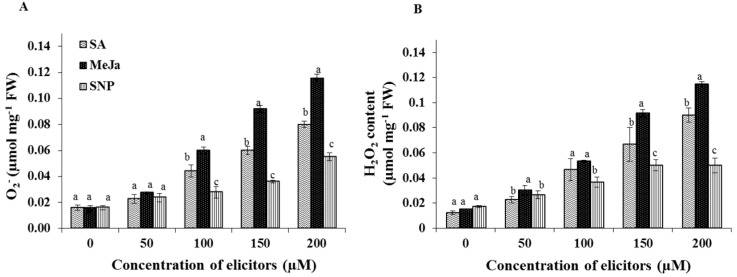
Effect of chemical elicitor treatments on the reactive oxygen species. Levels of superoxide (**A**) and endogenous hydrogen peroxide content (**B**) in cell suspension cultures. Data are the mean ± SE from three replicates. Different letters (a, b, c) in one measurement indicate statistically significant difference at *p* ≤ 0.05 by Duncan multiple range test.

The elicitor treatments significantly influenced the production of free radicals such as super oxide and hydrogen peroxide in the cell cultures. On the other hand, the un-elicited control cultures displayed the least amount of endogenous O_2^−^_ and H_2_O_2_ contents (Figure 3). Cells treated with MeJa, especially in 150 or 200 µM concentrations, displayed the maximum amount of O_2^−^_ accumulation (Figure 3A). However, the H_2_O_2_ content was significantly increased in both SA (150 or 200 µM) and MeJa (150 or 200 µM) treatments (Figure 3B). Both the levels of O_2^−^_ and H_2_O_2_ were less affected by different concentrations of SNP.

### 2.3. Effect of Chemical Elicitors on the Antioxidant Enzyme Activities of the Cells

**Figure 4:**
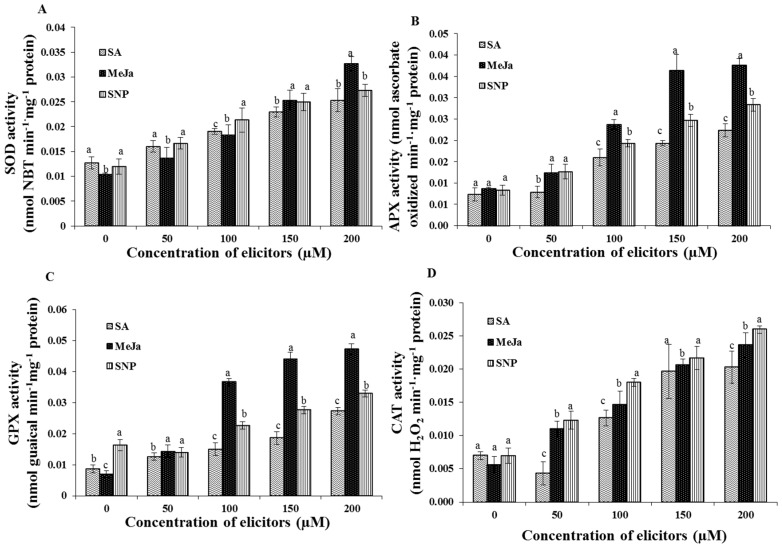
Effect of chemical elicitor treatments on antioxidant enzymes activities of cell suspension cultures. The activity of superoxide dismutase (**A**); ascorbate peroxidase (**B**); guaiacol peroxidase (**C**); and catalase (**D**) estimated in the cell suspension culture. Data are the mean ± SE from three replicates. Different letters (a, b, c) in one measurement indicate statistically significant difference at *p* ≤ 0.05 by Duncan multiple range test.

The analysis of antioxidant enzyme activities revealed that the cell cultures elicited with MeJa, SA, and SNP consisted of higher amounts of antioxidant enzymes activities in comparison with the control cultures (Figure 4). In accordance with the endogenous free radicals, the supplementation of MeJa particularly in a 200 µM concentration greatly increased the activities of superoxide dismutase (SOD), guaiacol peroxidase (GPX), and ascorbate peroxidase (APX) compare to the other elicitors. In detail, the greatest activity of SOD was observed in the MeJa at 200 µM concentration, whereas the application of SA and SNP elicitors increased the SOD activity without any significant differences (Figure 4A). In addition, activities of peroxidases (APX and GPX) were significantly enhanced by the MeJa treatments, especially in the 200 µM MeJa elicited cells (Figure 4B,C). However, the activity of catalase (CAT) was increased in the cells treated with increasing concentration of SNP treatments followed by the MeJa treatments (Figure 4D).

### 2.4. Effect of Chemical Elicitors on the Accumulation of Bioactive Compounds

**Figure 5:**
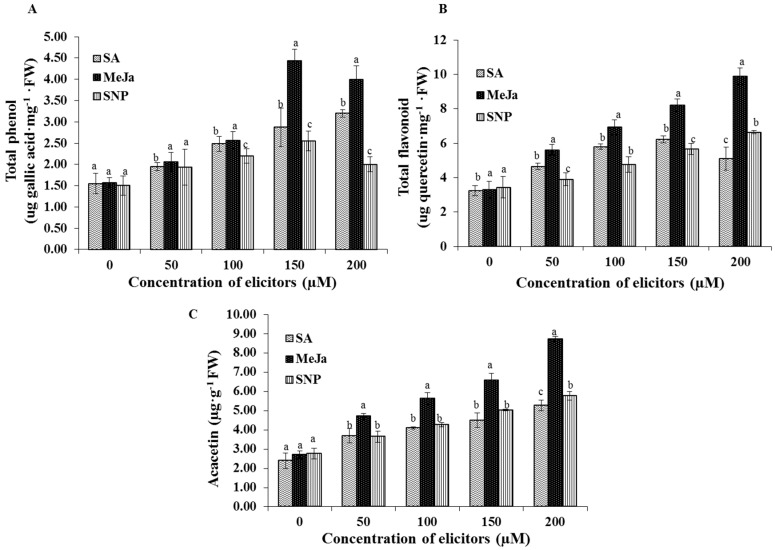
Effect of chemical elicitor treatments on the secondary metabolites. Total phenol content (**A**); total flavonoid content (**B**); and acacetin content (**C**) of cell suspension cultures. Data are the mean ± SE from three replicates. Different letters (a, b, c) in one measurement indicate statistically significant difference at *p* ≤ 0.05 by Duncan multiple range test.

The synthesis of bioactive compounds such as total phenols, total flavonoids, and acacetin were significantly influenced by the elicitor treatments (Figure 5). However, in contrast to the antioxidant enzyme activities, the highest concentration (200 µM) of MeJa and SNP slightly decreased the production of the total phenols. The greatest content of total phenols was noted in the cell extracts treated with the 150 µM MeJa (Figure 5A). On the contrary, the synthesis of total flavonoids was enhanced by the 200 µM MeJa treatment (Figure 5B). Similarly, the accumulation of the acacetin was elicited by the increasing concentrations of elicitors. Among the elicitors, MeJa significantly increased the acacetin content in the cell suspensions. The greatest content of acacetin was obtained in the cell suspension cultures elicited with the 200 µM MeJa more than the other elicitor treatments (Figure 5C).

### 2.5. Effect of Chemical Elicitors on the Free Radical Scavenging Potential of the Cell Extracts

**Figure 6:**
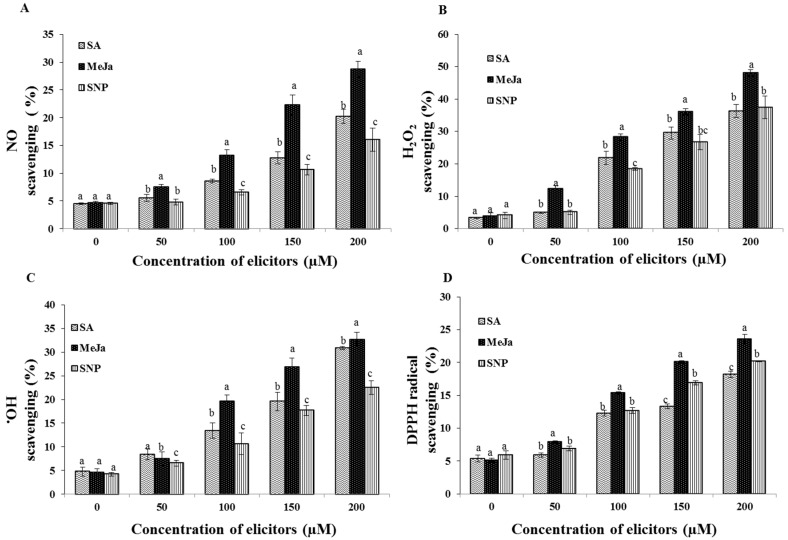
Effect of chemical elicitor treatments on free radical scavenging potentials of the extracts of cell suspension cultures. Nitric oxide scavenging potential (**A**); Hydrogen peroxide scavenging percentage (**B**); Hydroxyl radical scavenging capacity (**C**); DPPH radical scavenging potential (**D**) of cell extracts. Data are the mean ± SE from three replicates. Different letters (a, b, c) in one measurement indicate statistically significant difference at *p* ≤ 0.05 by Duncan multiple range test.

The chemical elicitation treatments significantly enhanced the free radical scavenging potential of the extracts of cell suspension culture in comparison with the control (Figure 6). The greatest NO scavenging potential of the cell extract (28.7%) was noted in the 200 µM MeJa treatment followed by SA in a concentration-dependent manner (Figure 6A). Similarly, the MeJa application in 200 µM increased the H_2_O_2_ scavenging potential of the cell extract to 48.1% than the other treatments (Figure 6B). However, the application of the SA and SNP elicitors displayed non-significant difference in the H_2_O_2_ scavenging potential. In accordance with the H_2_O_2_ scavenging, the extracts of cells elicited with the MeJa in 200 µM concentration resulted in the maximum ^•^OH scavenging potential of 32.6% followed by the 200 µM SA treatment (Figure 6C). Moreover, the greatest DPPH scavenging percent (23.6%) of the cell extracts was obtained in the 200 µM MeJa treatment followed by the 200 µM SNP treatment (Figure 6D).

Overall, the results suggest that the MeJa mediated-elicitation increases the accumulation of the bioactive compounds and the antioxidant potential than the SA and SNP in the cell suspension cultures of *S. kakudensis*.

## 3. Discussion

Plant-derived secondary metabolites are vital resources for naturaceuticals. However, the direct isolation of secondary metabolites from plants, and chemical synthesis of these compounds are cumbersome [16]. In order to overcome these difficulties cell suspension culture has been established in notable medicinal plants [17]. The extract of *S. kakudensis* is reported to encompass important secondary metabolites with pharmaceutical importance [12,13]. However, the lack of potential plant materials for the large-scale production of secondary metabolites hinders the utilization of *S. kakudensis*. Therefore, in the present study the cell suspension culture of *S. kakudensis* has been established. Callogenesis was achieved using leaf explants by the combination of BA and 2,4-D. According to a recent report, the addition of BA enhances the induction of callus in *S. kakudensis* particularly, at a 3.0 mg·L^−1^ concentration [13]. In the present experiment, the synthetic auxin 2,4-D was employed to induce the friable calli. However, no callus induction was observed in the PGR free medium.

The friable calli produced in the PGR medium were cultured in the liquid medium with continuous rotation to obtain the cell suspension. Remarkably, the proliferation of cells was accelerated in the liquid medium and the aggregation of granular cells was visible. Similarly, the friable calli displayed high frequency of cell proliferation upon transferring to the liquid medium in *Halodule pinifolia* [18]. Our results are in accordance with the previous studies reported on *Withinia somnifera* [19], *Psychotria carthogenensis* [20], and *Vetiveria zizanioides* [21]. Subsequent passage of the cells to new medium resulted in the formation of suspension with distinct single cells. The microscopic analysis of the cell suspension culture of *S. kakudensis* revealed the presence of single as well as clumps of cells. The morphological appearances of *S. kaudensis* cells were similar to the cell structures observed in *S. striata* [15] and *Halodule pinifolia* [18].

In order to investigate the effects of chemical elicitors, the cell suspension culture of *S. kakudensis* was elicited with MeJa, SA, and SNP. According to previous findings, the exogenous application of chemical elicitors mimics the response of a pathogen attack or wound signal, which triggers a defense response in plants by inducing the oxidative burst [15,22]. Correspondingly, the oxidative perturbation in the cells resulted in the accumulation of endogenous reactive oxygen species (O_2^−^_ and H_2_O_2_) in a concentration-dependent manner [23]. Among the elicitors, MeJa enhanced the production of O_2^−^_ and H_2_O_2_ in the cell suspension culture. In general, the MeJa is considered a potent plant signaling molecule which can modulate vital developmental processes and stress-related metabolism in plants [24,25,26]. The induction of ROS production upon exogenous application of MeJA has been evidenced in cell suspension culture of *Petroselinum crispum* [25]. According to Zhang and Xing [27], the induction of ROS increased in long-term MeJa treated protoplasts than the control cells. Moreover, the report illustrated that the H_2_O_2_ is vital for the MeJa signaling pathway, particularly with respect to programmed cell death (apoptosis) [27]. Moreover, the elicitor compounds MeJa and SA synergistically co-potentiated the production of ROS especially H_2_O_2_ in Arabidopsis and Tobacco [28]. In addition, the MeJa-treated plants displayed the occurrence of an oxidative burst leading to the activation of cascade of antioxidant metabolism in plants [28,29,30]. Moreover, the stimulation of H_2_O_2_ content by MeJa treatment resulted in the activation of defense signaling in tomato [31]. Thus, the oxidative stress induced by the elicitors triggered the antioxidant enzymes in order to detoxify the ROS in cell suspension cultures. In response to stress conditions, plants activate the antioxidant enzymes such as SOD, CAT, APX, and GPX. During the ROS detoxification process, the primary reaction was catalyzed by the SOD. This enzyme provides the first line of defense against the toxic effects of elevated levels of ROS [29]. The SOD enzyme catalyzes the dismutation of O_2^−^_ into H_2_O_2_ and O_2_. Subsequently the H_2_O_2_ is scavenged by CAT and/or peroxidases such as GPX and APX into H_2_O and O_2_ [29].

The elicitors increased the accumulation of major antioxidant compounds such as total phenols and total flavonoids in the cell suspension cultures of *S. kakudensis*. In general, the elicitor compounds positively influence the transcriptional regulation of genes of the vital enzymes involved in the phenylpropanoid pathway. For instance, the up-regulation of phenylalanine ammonia lyase (PAL) m-RNA transcripts by MeJa, SA, and SNP have been reported previously [6,24,31,32,33,34]. Hence, the predominant antioxidant compounds such as phenols and flavonoids are synthesized via the phenylpropanoid pathway, the stimulation of the important enzymes in the pathway result in the accumulation of secondary metabolites. Although the elicitor molecules increase the activity of thenphenylpropanoid pathway, the effect of the elicitor molecules varies between plant species. However, in *S. kakudensis*, MeJa significantly enhanced the production of total phenols and total flavonoids. Likewise, the MeJa elicited the synthesis of polyphenolic compounds and the flavonol content in the *Vitis vinifera* cell cultures [3].

Moreover, the higher production of endogenous ROS significantly correlated with the secondary metabolite content. Similarly, the higher-concentration elicitors significantly elicited the synthesis of acacetin in the cell culture. Acacetin is a therapeutically important flavonoid compound present in the *S. kakudensis*. The present investigation is the first report that deals with the effect of chemical elicitors on the accumulation of acacetin. The induction of genes related to the flavonoid biosynthesis by the elicitors could be the possible rationale behind the elicitation of acacetin in cell suspension culture [33]. Similarly, the activation of genes responsible for the synthesis of flavonoids was observed in *Glycine max* [34], *Taxus cuspidata* [35], and Petunia [36]. In *Panax ginseng*, the root cultures treated with MeJa and SA increased the production of ginsenosides [29].

Antioxidants such as phenols and flavonoids prevent from the excess production of free radicals in the cells. Among the free radicals, H_2_O_2_, ^•^OH, and NO, are the most harmful radicals to the cells [37]. Eventually, the increases in the levels of free radicals result in cell death by oxidation of the bio-macromolecules such as protein, DNA, and unsaturated fatty acids in humans and animals [38]. Therefore, it is necessary to evaluate the ability of the *S. kakudensis* cell extracts to combat free radicals. According to the present results, both the elicited and the control cell extracts possessed free radical scavenging potential. However, the cell extracts obtained from the elicitor-treated cells displayed higher free radical scavenging activities. Taken together, the elicitation of bioactive secondary metabolites by the chemical elicitors improved the free radical scavenging potentials of the cell extracts.

## 4. Experimental Section

### 4.1. Plant Materials and Culture Conditions

Leaf explants were excised from the *in vitro* shoots of *S. kakudensis* and inoculated on the Murashige and Skoog (MS) [39] basal medium with 3% (*w*/*v*) sucrose and 0.8% (*w*/*v*) agar containing plant growth regulators. For friable callus induction, 3.0 mg·L^−1^ 6-benzyladenine (BA) was employed based on the previous report [13] in combination with 0.5 mg·L^−1^ 2,4-dichlorophenoxyacetic acid (2,4-D). The pH of all the media used in this experiment was adjusted to 5.8 before autoclaving at 121 °C for 15 min. All cultures were maintained at 25 °C and 80% RH under a 16 h photoperiod with 50 μmol·m^−2^·s^−1^ PPFD provided by cool white fluorescent light (40 W tubes, Philips, The Netherlands).

### 4.2. Establishment of Cell Suspension and Elicitor Treatments

The friable callus induced from the leaf explant was transferred to liquid MS medium (100 mL) containing 3.0 mg·L^−1^ BA along with 0.5 mg·L^−1^ 2,4-D in 200 mL Erlenmeyer flask. The cultures were maintained under continuous shaking with 120 rpm under 16 h photoperiod with 50 μmol·m^−2^·s^−1^ PPFD provided by cool white fluorescent light (40 W tubes, Philips, The Netherlands) in a rotary shaking incubator (KSI-200L, Koencon, Hanam, Korea). The cell suspension cultures were subcultured for every three week for 12 weeks, until the desired amount of suspensions were acquired for elicitation. For elicitation treatments, elicitors such as methyl jasmonate (MeJa), salicylic acid (SA), and sodium nitroprusside (SNP) in 50–200 µM concentrations were added to the medium. The cell cultures were maintained in the elicitor treatment for two weeks before harvesting. The cell cultures without elicitor treatment were considered controls. All treatments were conducted with three biological replicates and the experiment was conducted in the randomized block design.

### 4.3. Microscopic Observation of Cells

For microscopic observation, cells were stained with 0.01% toluidine blue and observed under 10× and 40× magnifications using a light microscope (Y-TV55, Nikon, Tokyo, Japan).

### 4.4. Estimation of Superoxide (O_2^−^_) and Hydrogen Peroxide (H_2_O_2_)

For superoxide estimation, samples (0.1 g) were lyophilized and mixed with 0.5 mL of 65 mM phosphate buffer (pH 7.8). The homogenate was centrifuged at 5000× *g* for 10 min at 4 °C. The supernatant (0.1 mL) was mixed with 10 mM hydroxylamine chlorohydrate (0.02 mL) and 65 mM phosphate buffer (pH 7.8) and incubated at room temperature for 20 min. After the incubation, the mixture was combined with 17 mM sulfanilamide (0.02 mL) and 7 mM α-napthylamine and again incubated in room temperature for 20 min. To prevent the chlorophyll interference, ether (0.6 mL) was added to the mixture and centrifuge at 10,000× *g* for 15 min at 4 °C. The absorbance of the supernatant was measure at 530 nm and the superoxide content was estimated using the standard sodium nitrite calibration curve. The endogenous H_2_O_2_ level was measured at 390 nm using the standard H_2_O_2_ calibration curve [40].

### 4.5. Estimation of Antioxidant Enzyme Activities

To determine the antioxidant enzymes activity, 0.1 g of vaccum-dried cells were homogenized in 50 mM phosphate buffer (pH 7.0) containing 1 mM EDTA, 0.05% triton X, and 1 mM polyvinylpyrolidone (PVP). Then the homogenate was centrifuged at 10,000× *g* for 20 min at 4 °C and the supernatant was used for determination of antioxidant enzymes activity. Superoxide dismutase (SOD), catalase (CAT), ascorbate peroxidase (APX), and guaiacol peroxidase (GPX) enzyme activities were estimated by following the protocols of Manivannan *et al.* [40]. The total protein content was estimated at 595 nm according to Bradford method [41] using bovine serum albumin as standard.

### 4.6. Extract Preparation for Phytochemical Analysis

The cells were harvested using 0.45 µM sieve filters and the excess medium was vacuum dried. For phytochemical and free radical scavenging assessment, control and elicitor treated suspensions were extracted with methanol. Briefly, the cells (0.1 g) were lyophilized and extracted with 1 mL of 80% (*v*/*v*) methanol for overnight under 150 rpm in a rotating shaker. The resulting homogenates were centrifuged at 10,000× *g* for 10 min and the supernatant was employed for the *in vitro* assays [13].

### 4.7. Estimation of Bioactive Compounds

#### 4.7.1. Estimation of Total Phenols and Flavonoids

For total phenol estimation, aliquot of the extracts (0.1 mL) made up to 1 mL with distilled water was mixed with 0.5 mL of Folin-Ciocalteu reagent (1:1 with water) and 2.5 mL of sodium carbonate solution (7.5%). The reaction mixture was vortexed vigorously and incubated in dark for 40 min. After incubation the absorbance was recorded at 725 nm and the total phenol content was expressed as gallic acid equivalents (GAE) [11]. The total flavonoid composition was determined based on the aluminum chloride calorimetric method. Samples (0.1 mL) were made up to 1 mL with 80% methanol and used for the analysis by adding 1 mL of 2% aluminum chloride solution. The absorbance of the reaction mixture was measured at 415 nm after 30 min incubation and the total flavonoids were calculated from the standard quercetin calibration curve [13].

#### 4.7.2. Quantification of Acacetin Using High Performance Liquid Chromatography (HPLC)

The plant extract preparation and estimation of acacetin was carried out according to the procedure outlined by Yang *et al.* [42]. Briefly, the samples (1.0 g) were lyophilized and refluxed in 50 mL methanol for 24 h in a rotatory shaker at 150 rpm and concentrated under reduced pressure. The concentrated extracts were filtered in a 0.45 µm syringe filter prior to chromatographic analysis in a 1200 series HPLC instrument with diode array detector (DAD) (Waters, MA, USA). The mobile phase consisted of 100% acetonitrile (solvent A) and 1.0% glacial acetic acid (solvent B). The chromatographic separation was performed with solvent proportion of 37:63 using an Hypersil ODS column (Thermo Fischer Scientific, MA, USA) (4.6 × 250 mm, 5 µM) with 1.25 mL·min^−1^ flow rate with 10 µL sample injection volume. The absorbance of the standards and samples were recorded at 326 nm. The quantity of acacetin content was elucidated from the standard calibration curve. The limit of detection (LOD) and limit of quantitation (LOQ) values were determined from the standard curve. An HPLC chromatogram of the acacetin in reference and the cell extract along with LOD and LOQ values have been provided in the supplementary material Figure S1.

### 4.8. Assessment of Free Radicals Scavenging Potential of Cell Extracts

#### 4.8.1. Superoxide (O_2^−^_) Radical Scavenging Assay

Superoxide scavenging activity of the extracts was determined based on the ability of the extracts to inhibit formazan production by bleaching the superoxide radicals generated by nitroblue tetrazolium salt with riboflavin and light. The extracts (0.1 mL) were added to the reaction mixture (0.1 mg NBT, 12 mM EDTA, and 20 µg riboflavin in 50 mM sodium phosphate buffer (pH 7.6)) and illuminated by light. After 90 s the absorbance was measured at 590 nm [37].

#### 4.8.2. Nitric Oxide (NO) Radical Scavenging Assay

In this assay the inhibition of NO production by the extracts was determined using sodium nitroprusside (SNP) mediated generation of nitric oxide. The nitric oxide spontaneously produced by SNP reacts with the oxygen to form nitrite ions that can be measured using a Griess reagent. The reaction was initiated by the addition of 10 mM SNP in phosphate-buffered saline to the extracts (0.1 mL) and allowed to stand for 150 min in room temperature. After incubation, 0.5 mL of freshly prepared Griess reagent (2% phosphoric acid, 1% sulfanilamide, and 0.1% *N*-(1-napthyl) ethylenediamine dihydrochloride) was added and the absorbance was determined at 546 nm [37].

#### 4.8.3. Hydrogen Peroxide (H_2_O_2_) Radical Scavenging Assay

For H_2_O_2_ scavenging assay, 0.6 mL of H_2_O_2_ (2 mM) was mixed with the extracts and incubated for 10 min. The absorbance was noted at 230 nm against a blank solution devoid of H_2_O_2_ according to the method described by Kumaran and Karunakaran [37].

#### 4.8.4. 2,2-Diphenyl-1-picrylhydrazyl (DPPH) Radical Scavenging Assay

The stable DPPH radical scavenging ability of the extracts was analyzed by mixing sample extracts (40 µL) to 1960 µL of 0.1 mM methanolic solution of DPPH and allowed to stand for 25 min under dark conditions. The absorbance of the sample was measured at 517 nm [13].

For all the free radical scavenging assay ascorbic acid was employed as the standard. The radical scavenging % was calculated using the formula [(*A*_c_ − *A*_s_)/*A*_c_] × 100 where *A*_c_ is the absorbance value of the control (reaction mixture without extract) and *A*_s_ is the OD value of the extract or ascorbic acid.

All the chemicals used for the elicitation treatments, phytochemical analysis, and antioxidant potential assessment were of analytical grade purchased from Sigma Aldrich (St. Louis, MO, USA).

### 4.9. Statistical Analysis

The treatments were set up in a completely randomized design with three replications per treatment. Significant differences among the treatments were determined by analysis of variance (ANOVA) followed by Duncan’s multiple range tests at a significant level of *p* ≤ 0.05 (*n* = 3) using Statistical Analysis System (SAS, V.6.12) computer package (SAS Institute Inc., Cary, NC, USA). The Pearson correlation co-efficients of the endogenous ROS and secondary metabolites were provided in the supplementary material Table S1.

## 5. Conclusions

In conclusion, the cell suspension culture system has been successfully established for *S. kakudensis*. The elicitor treatments significantly modulated the accumulation of endogenous O_2^−^_ and H_2_O_2_, which in turn activated the antioxidant metabolism in cells. Eventually, the perturbation in the antioxidant mechanism increased the synthesis of bioactive phytochemicals such as total phenols and total flavonoids. In addition, the MeJa treatment significantly elicited the acacetin content in the cell suspension culture. The improvement in the levels of phytochemicals enhanced the free radical scavenging ability of the cell suspension extracts. Therefore, the outcomes of the present study can be utilized for the large-scale production of bioactive compounds in *S. kakudensis*.

## Supplementary Materials

Supplementary materials can be found at http://www.mdpi.com/1422-0067/17/3/399/s1.
