# Supplementary material for: Chemical Elicitor-Induced Modulation of Antioxidant Metabolism and Enhancement of Secondary Metabolite Accumulation in Cell Suspension Cultures of Scrophularia kakudensis Franch
Source: Int J Mol Sci. 2016 Mar 18;17(3):399. doi: 10.3390/ijms17030399 (PMC4813254; doi:10.3390/ijms17030399)
Supplement: Supplementary file 1 [file ijms-17-00399-s001.pdf]

Supplementary Materials: Chemical Elicitor-Induced Modulation of Antioxidant Metabolism and Enhancement of Secondary Metabolite Accumulation in Cell Suspension Cultures of *Scrophularia kakudensis* Franch

Abinaya Manivannan, Prabhakaran Soundararajan, Yoo Gyeong Park and Byoung Ryong Jeong

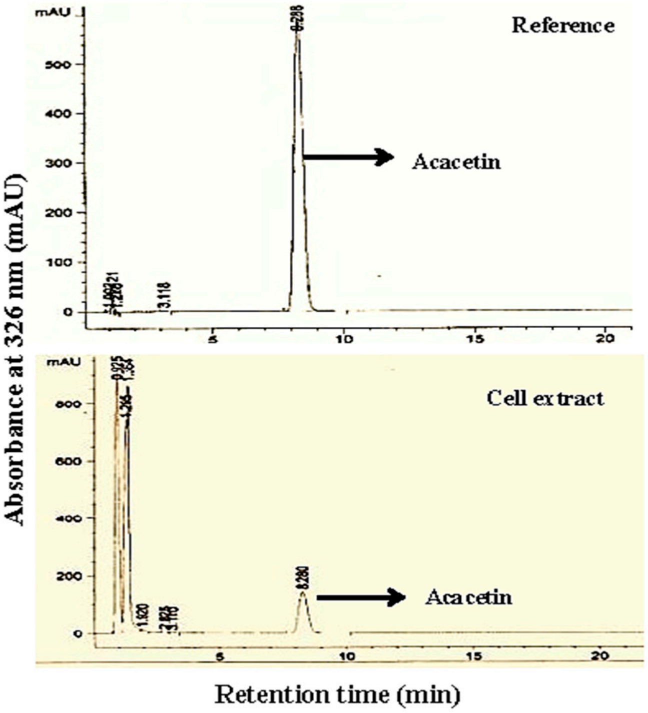

Figure S1. HPLC chromatograms of acacetin—reference and cell extract along with the LOD and LOQ values. Limit of detection (LOD) = 0.19 μg·mg^{-1}; Limit of quantitation (LOQ) = 0.65 μg·mg^{-1}.
